# Bridging the Evidence-to-Policy Gap: Strengthening Capacities in Low- and Middle-Income Countries to Translate Antimicrobial Resistance Data and Evidence into Effective Policies

**DOI:** 10.3390/antibiotics15030255

**Published:** 2026-03-01

**Authors:** Prerana Parajulee, Sajan Gunarathna, Anthony Burnett, Jae Hee Hwang, Jung-Seok Lee, Fadi El-Jardali, Satyajit Sarkar

**Affiliations:** 1 Policy and Economic Research (PER) Department, Epidemiology Public Health Impact (EPIC) Unit, International Vaccine Institute (IVI), Seoul 08826, Republic of Korea; 2 Department of Health Management and Policy, Faculty of Health Sciences, American University of Beirut, Beirut 1107 2020, Lebanon; 3 Knowledge to Policy Center, Faculty of Health Sciences, American University of Beirut, Beirut 1107 2020, Lebanon; 4 Department of Health Research Methods, Evidence and Impact, McMaster University, Hamilton, ON L8N 3Z5, Canada

**Keywords:** antimicrobial resistance, policy, evidence brief for policy, low and middle-income countries

## Abstract

**Background:** The translation of antimicrobial resistance (AMR) data and evidence into policy remains limited in many low- and middle-income countries (LMICs) in Asia and Africa, despite expanded investments being made in AMR surveillance and research. This is due to fragmented governance, weak knowledge translation capacity, and insufficient multisectoral coordination. **Results**: Following the implementation of an Evidence-to-Policy (E2P) capacity-strengthening intervention in four LMICs in Africa and Asia, participant surveys showed improved confidence and capability to synthesize, interpret, and apply AMR evidence to inform policy. The four countries highlighted persistent constraints such as sectoral silos, limited institutional ownership, and gaps in evidence-use systems, but reported enhanced cross-sectoral collaboration and a clearer, replicable process for the development of Evidence Briefs for Policy (EBPs). **Methodology:** During Phase II of the Fleming Fund-resourced Regional AMR Data Analysis for Advocacy, Response, and Policy (RADAAR) project, a structured, hybrid, evidence-to-policy (E2P) capacity-strengthening model was implemented in Bhutan, Ghana, Kenya, and Lao People’s Democratic Republic, combining online and in-person training, targeted mentorship, and policymaker engagement. Each country developed a country-specific evidence brief for policy (EBP) and conducted policy dialogues to facilitate stronger decision maker involvement. **Conclusions**: RADAAR’s approach strengthened the foundational capacity for evidence-informed policymaking and demonstrated the value of institutionalized knowledge translation mechanisms. Sustained investment in E2P systems is essential to bridge the AMR E2P gap and ultimately support AMR prevention and control.

## 1. Introduction

Antimicrobial resistance (AMR) is a critical global health threat, with low- and middle-income countries (LMICs), particularly in Asia and Africa, bearing the brunt of its impact [1,2]. These settings are marked by a high prevalence of infectious diseases, insufficient diagnostic infrastructure, and a shortage of skilled healthcare professionals [3,4]. Initiatives to generate policy-relevant AMR data are prone to persistent systemic and resource-related constraints, including fragmented surveillance systems, inconsistent laboratory standards, and uneven data coverage, which hinder the availability of reliable evidence to guide effective interventions. Although high-quality data are being generated by research institutions, governments, non-governmental organizations (NGOs), and international non-governmental organizations (INGOs), their translation into policy and decision making remains limited, with evidence frequently confined to reports or scientific publications rather than being translated into policies for impact [5,6,7].

Evidence from the Tracking AMR Country Self-Assessment Survey (TrACSS) showed that in many LMICs, AMR data are not being systematically analyzed or used to inform policy development, resource allocation, or operational decision making, with little improvement observed over time [8].

**Figure 1:**
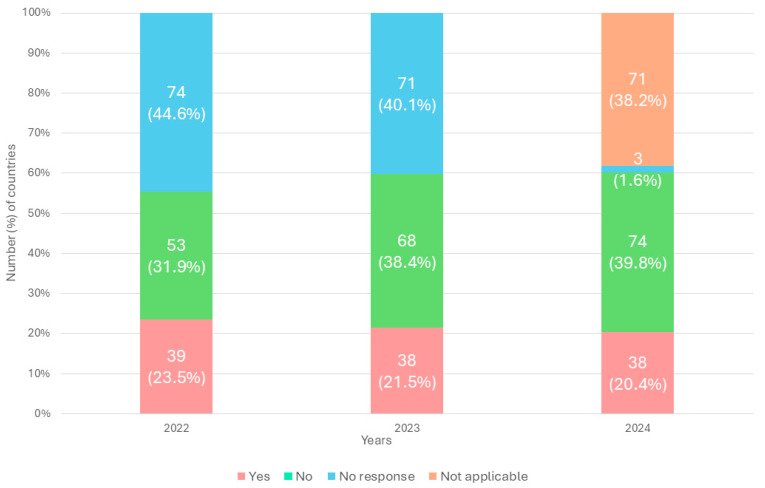
Country response to use of data for decision making and policy change TrACSS (2022–2024). N/A denotes not applicable, which follows the TRACSS definition and indicates that the question was not triggered by the survey logic and therefore was not presented to respondents.

Between 2022 and 2024, only a small and largely unchanged proportion of countries reported the systematic use of AMR data for policymaking (39/166 in 2022, 38/177 in 2023, and 38/186 in 2024). In contrast, the number of countries reporting “no use” of AMR data increased steadily over the same period (53/166 in 2022, 68/177 in 2023, and 74/186 in 2024). Although missing responses declined, collectively, a substantial proportion of countries continued to report “no use” or “not applicable” responses in 2024 (Figure 1). These trends highlight a persistent gap between AMR data generation and its application in policy processes, underscoring the need for structured and sustained support to strengthen national capacities for translating AMR evidence into effective and actionable policies [9,10].

The 2022 to 2024 period reflects the official RADAAR implementation timeline. Accordingly, our analysis focused on activities and policy processes supported during this cycle. This does not suggest that AMR policymaking began in 2022, but rather that the scope was limited to the defined project period for consistency and alignment with its objectives.

The Regional Antimicrobial Resistance Data Analysis for Advocacy, Response, and Policy (RADAAR) project was implemented in response to this evidence–policy gap. The RADAAR project strengthens regional capacities for collecting, analyzing, and translating AMR data into actionable policy across human, animal, and environmental health sectors. The project led by the Policy and Economic Research Department of the International Vaccine Institute (IVI) and funded by the Fleming Fund (FF) adopted a structured, capacity-building approach informed by an analysis of country reported TRACSS responses. This approach combined targeted training, sustained mentorship, the development of evidence briefs for policy (EBPs), and structured policy dialogues to support the institutionalization of evidence-informed decision making in LMICs.

Thus, based on the RADAAR experiences across selected countries in Asia and Africa, this paper presents key lessons, practical strategies to improve data uptake, and recommendations to strengthen national systems for linking AMR evidence to policy.

## 2. Results

### 2.1. Pre- and Post-Training Survey Assessment

To assess the extent to which participants capacity, we conducted pre- and post-training surveys for both the in-person and online sessions. The surveys included structured yes/no items across key thematic areas, followed by open-ended questions to gather additional contextual insights. The analysis was descriptive. Quantitative responses were summarized using frequencies and proportions, while open-ended responses were reviewed and grouped thematically to identify recurring patterns. These surveys explored shift in participants understanding, skills, and confidence in applying evidence-informed policymaking tools. The pre-training survey was completed by 43 participants, and the post-training survey by 36 participants.

Results showed that over 91.7% (33/36) of participants recognized the value of multisector engagement, 63.9% (23/36) identified new opportunities to apply the evidence brief for policy (e.g., AMR regulation, health insurance, stewardship), and 94.4% (34/36) felt confident in interpreting and applying evidence. Although challenges in accessibility and dissemination remained, these were expected to improve through planned policy dialogues and subsequent follow-up support. Overall, these findings indicate improvement in participants self-reported capacity to apply evidence in policymaking (Table 1).

Compared to the previous fully online model in Phase I of the RADAAR project, the hybrid approach in Phase II facilitated deeper engagement, increased country ownership, and strengthened stakeholder capacity. These improvements were supported by pre- and post-training assessments that demonstrated measurable improvements in participants’ understanding and application of evidence-to-policy tools.

### 2.2. Country Reflections on AMR Evidence-to-Policy Strengthening and RADAAR

The surveys were conducted immediately before the initiation of the in-person or online training sessions and within days of completing the seven module lecture series. As a result, the evaluation timeframe was limited to short-term outcomes and did not allow assessment of longer-term effects. To complement the survey data, we collected qualitative feedback through written responses from participants actively engaged throughout the training and EBP development process.

The following quoted perspectives reflect each country’s overall view of its progress in AMR evidence-to-policy processes, as well as the role of RADAAR in addressing national-level gaps.

### 2.3. Bhutan

*“In Bhutan, the availability of local data, research capacity, and competing priorities have shaped the use of AMR evidence in policymaking. In response, the country team engaged decision-makers from the human and animal health sectors and promoted AMR topics through national forums, helping to increase awareness and policy interest.”*

*“With support from RADAAR, national capacity was strengthened through training, mentorship, and the development of an Evidence Brief for Policy. Policy dialogues supported engagement with senior Ministry of Health officials on issues related to multidrug-resistant organisms and healthcare-associated infections. Further work, including costing and feasibility assessments and continued technical engagement, will support sustained progress.”*

### 2.4. Ghana

*“In Ghana, competing priorities, coordination across One Health sectors, and communication gaps between researchers and policymakers influenced the use of AMR data for decision-making. In response, an AMR platform and an AMR Secretariat were established to strengthen coordination and support the dissemination of research findings through platform and Technical Working Group meetings. In addition, cost and output studies were conducted to support advocacy for increased resource allocation for AMR.*

*Through RADAAR, training and mentorship in developing Evidence Briefs for Policy strengthened individual capacity and cross-sector collaboration. As policy development continues, ongoing policy dialogue and stakeholder engagement are expected to further support AMR prioritization.”*

### 2.5. Kenya

*“In Kenya, the use of AMR data for policymaking is influenced by factors such as the organization of data systems, variability in laboratory capacity, and the need for continued coordination across sectors and levels of the health system. Ongoing efforts to strengthen data integration and analytical capacity have contributed to improved availability and use of AMR evidence.”*

*“Support from RADAAR has contributed to capacity strengthening through training, mentorship, and policy dialogues, facilitating the development of policy-relevant AMR outputs and encouraging cross-sector collaboration within a One Health framework. Continued investment in capacity building, coordination mechanisms, and sustainable financing will help maintain and build on this progress.”*

### 2.6. Lao PDR

*“In Lao PDR, inter-ministerial coordination, laboratory capacity, and funding arrangements have influenced the use of AMR evidence in policymaking. While a costed National Strategic Plan on AMR (2026–2030) has been developed and coordination mechanisms strengthened, resource and implementation constraints remain.*

*With support from RADAAR, the development of an Evidence Brief for Policy on AMR awareness supported engagement with decision-makers. Workshops and mentoring strengthened national capacity to synthesize evidence and develop policy-relevant outputs. Continued investment, strengthened implementation mechanisms, and sustained technical support will help build on these gains.”*

In addition to the four countries, we collaborated closely with Nigeria on the development of their evidence brief for policy (EBP).

### 2.7. Implementation Considerations Identified Across EBPs

Analysis of the evidence briefs for policy (Table 2) identified both cross-cutting and country-specific implementation considerations. Across countries, common constraints included limited domestic financing for AMR activities, competing health and development priorities, and regulatory or policy approval bottlenecks that could delay implementation. EBPs also highlighted challenges related to sustainability of funding beyond external donor support.

In contrast, country-specific implementation hurdles varied according to context. These included procurement and supply chain logistics, workforce and laboratory capacity limitations, and coordination gaps across One Health sectors or administrative levels. Some countries emphasized inter-ministerial coordination challenges, while others highlighted data integration constraints or sector-specific regulatory complexities.

### 2.8. Policy Dialogue and Key Outputs

Structured, multistakeholder policy dialogue events were conducted in the four focus countries engaging key decision makers across AMR and related sectors. These dialogues facilitated evidence-based policy discussions and resulted in concrete commitments to AMR control, while ensuring strong national ownership. Lessons generated from these dialogues were shared with other countries to inform similar efforts.

The key outputs included the following:

- Development of an EBP in the four focus countries and along with additional EBPs in interested countries.
- Strengthening capacity of multisectoral stakeholders through online and offline training to interpret and apply AMR evidence in policy processes.
- Engagement of policymakers, researchers, and program managers via in-country policy dialogues, fostering their involvement in policy uptake.

## 3. Discussion

The discussion primarily draws on Phase II participant feedback surveys EBPs developed by the four focus countries and selected findings from Phase I focus group discussions and key informant surveys [11,12,13,14,15].

Country selection was partly informed by existing collaborative relationships with implementing partners. Leveraging established networks allowed the project to build on pre-existing trust, coordination mechanisms which are recognized determinants of effective implementation and evidence uptake. This was particularly important given the relatively short implementation timeframe. Establishing new partnerships would likely have required substantial time for stakeholder alignment and trust building, potentially limiting the feasibility of delivering the full EBP development and policy dialogue process within the project period. However, working within established networks may limit generalizability to settings without similar collaborative infrastructure, where additional time and resources may be required to achieve comparable outcomes [16,17].

### 3.1. Availability of AMR Data

Over the past decade, national and regional surveillance systems have strengthened the availability and quality of AMR data across LMICs and high-income countries (HICs) [6]. Improvements in laboratory capacity, expansion of digital reporting platforms, and integration of AMR surveillance into routine health surveys have contributed to more reliable and comprehensive datasets [18,19]. Notable initiatives, including the WHO’s Global AMR and Use Surveillance System (GLASS) and FF regional and country grants, have supported these efforts [20,21].

### 3.2. Barriers to Policy Uptake

Despite these gains, several systemic and operational barriers continue to impede the effective use of AMR data in policy and planning. Fragmented governance and sectoral silos among human, animal, food, and environmental health continue to hinder the implementation of unified One Health strategies [22,23]. Limited capacity for policy translation, lack of institutional ownership, and absence of dedicated knowledge translation platforms further constrains the use of evidence in policy development [24,25,26]. Political, institutional, and economic challenges, including inconsistent leadership, competing health priorities, and insufficient funding, further limit AMR prioritization at the national level [27,28].

Additionally, a disconnect persists between researchers and policymakers: research outputs are often academically oriented and not sufficiently tailored to decision makers’ needs, widening the evidence-to-policy gap [29]. Survey findings from the online module highlighted these barriers, showing that while 87% of participants engaged across multiple sectors, only 52% reported policy uptake of recommendations, with limited accessibility, government endorsement, and dissemination (39%, 35%, and 30%, respectively) (Table 1).

Country-level assessments further identified contextual obstacles. In Bhutan, these obstacles include limited IT infrastructure, weak data integration, resistance to digital tools, sociocultural practices affecting IPC, and concerns about data privacy and electronic monitoring systems [30,31]. In Ghana, examples include pressure for inappropriate antibiotic use, weak regulatory enforcement, inconsistent protocol adherence, supply chain barriers, and limited legal mandates [32,33]. In Kenya, fragmented surveillance systems, uneven laboratory coverage, and capacity constraints across national and county levels have influenced the use of AMR evidence in policymaking [34,35]. In Lao PDR, obstacles include limited community-specific research, financial constraints driving pharmacy-based care, poor adherence to veterinary instructions, intense pharmaceutical marketing, and dependence on external technical support [36,37].

### 3.3. Challenges, Achievements, and Lessons Learned

While these barriers are well documented, Phase II of RADAAR was designed to address them through an applied mentoring model rather than training alone. In contrast to Phase I, which was delivered entirely online within limited timeframe, Phase II combined structured in-person training with sustained technical mentorship throughout the EBP development process.

This transition from knowledge acquisition to guided application strengthened policy translation. Mentors worked closely with country teams in topics aligned with national priorities, ensured adherence to standardized EBP methodology, and provided iterative feedback on draft briefs. The continuous review process enhanced analytical quality, clarity, and policy relevance, enabling teams to produce decision-ready output rather than academically oriented documents.

The findings from the RADAAR project suggest that the mentoring model directly addressed identified barriers. Iterative document review helped reframe technical content into concise briefs tailored to policymakers. Structured stakeholder mapping strengthened multisector engagement within a One Health framework. Refinement of recommendations improved feasibility by aligning proposals with regulatory, financial, and implementation realities [38,39,40]. Countries highlighted (Table 2) persistent challenges in translating AMR evidence into policy during the EBP development and dialogue preparation process. Sectoral fragmentation, where stakeholders often work in silos rather than adopting a unified One Health approach and limited inter-ministerial coordination, remains a major challenge consistent with findings from recent global assessments [41,42]. Studies have similarly noted weak multisectoral governance and under-resourced or poorly institutionalized co-ordination mechanism, limiting policy coherence [43]. Even where surveillance data are available, weak evidence-use systems and misalignment between research outputs and policymakers need continue to constrain translation into actionable policy [44,45,46]. However, within the RADAAR mentoring framework, these structural constraints were addressed through hands-on technical accompaniment rather than training alone, enabling teams to work through real-time bottlenecks during document development and dialogue preparation.

Operational challenges included competing priorities and high workload among health staff, inconsistent engagement across time zones, and project delays particularly from delays in obtaining timely approvals [47,48,49,50]. Despite these constraints, the structured mentorship model provided continuity and accountability, helping maintain progress and adherence to agreed timelines.

Compared with the earlier online-only model, the hybrid mentoring approach resulted in deeper engagement and stronger country ownership. Sustained technical guidance throughout the EBP development process improved methodological consistency, strengthened analytical quality, and enhanced the policy relevance of outputs.

Nevertheless, the RADAAR training and mentorship were widely viewed as highly valuable for building local capacity and strengthening collaboration across One Health sectors. Policy dialogues effectively engaged ministerial stakeholders, advanced national AMR priorities, and, in several cases, contributed to concrete policy commitments. In Bhutan, engagement with high-level AMR decision makers resulted in Ministry of Health commitments to AMR and hospital acquired infection (HAI) strategies. In Ghana, the initiative strengthened EBP development capacity, improved cross-sector coordination, and supported structured policy dialogues. In Kenya, the initiative strengthened the capacity to synthesize and translate AMR evidence, supported the development of policy-relevant outputs, and enhanced cross-sector engagement in AMR decision making. In Lao PDR, the process provided an effective entry point for broader AMR related engagement with decision makers. Overall, the initiative demonstrated the value of combining technical capacity building with structured policy engagement to support sustained evidence-informed policymaking.

Although no additional phase is planned under the Fleming Fund, lessons from Phase II provide a foundation for future AMR evidence-to-policy capacity strengthening. Early involvement of multisectoral stakeholders fosters stronger ownership of the output. Combining online and in-person training with follow-up mechanisms enhances flexibility and sustainability. Structured policy dialogues aligned with national AMR priorities were particularly effective in generating momentum for the uptake of evidence briefs, while regional and global partnerships further strengthened national efforts [50,51,52,53,54].

### 3.4. Limitations

While the RADAAR project demonstrated clear benefits, some limitations need to be acknowledged.

- Comprehensive technical support was provided to only four focus countries, while others received partial or ad hoc assistance, potentially limiting the overall impact.
- The relatively short project timeframe constrained assessment of longer-term outcomes, which may only become evident over time.
- Logistical and duration-related constraints limited deeper engagement with a broader set of countries.

### 3.5. Recommended Policy Implications Derived from the RADAAR Experience

To enhance uptake, countries emphasized the importance of co-developing evidence briefs and policy dialogues tailored to decision makers, investing in policymaker capacity-building, and leveraging regional platforms for knowledge exchange. Sustained communication, feedback mechanisms, and dedicated knowledge translation platforms were identified as critical enablers for bridging the evidence-to-policy gap [48,50,55,56,57,58].

Key policy recommendations include the following:

- Establishing a permanent platform to institutionalize evidence-informed AMR policy generation.
- Embedding standardized policy development within national planning processes.
- Strengthening technical capacity to translate available evidence into actionable policy.
- Ensuring structured coordination between technical teams, including surveillance and other data generating units, and the policy departments.
- Promoting a culture of One Health approach through sustained multisectoral engagement.
- Securing sustained funding to avoid interruptions in AMR initiatives.

A dedicated knowledge translation platform (KTP) can play a central role in operationalizing these policy recommendations by providing a structured and institutionalized mechanism to translate AMR evidence into decision-ready policy options. In practical terms, such a platform could be formally embedded within existing national AMR coordination structures, such as the National AMR Steering Committee or Ministry of Health policy units, to ensure institutional ownership. Governance could include a multisectoral oversight committee representing human, animal, environmental, and food sectors, supported by technical working groups responsible for evidence synthesis, economic analysis, and policy drafting. A small, dedicated secretariat or coordination unit could manage routine operations, including commissioning rapid evidence reviews, organizing structured policy dialogues, and maintaining communication channels between researchers and decision makers. Clear reporting lines to senior ministry leadership and integration into national planning and budgeting cycles would help ensure sustainability and policy relevance.

By systematically synthesizing and contextualizing data from surveillance, research, and programmatic sources, KTP would support consistent use of evidence in national policy processes rather than ad hoc or project-based engagement. Importantly, such a platform would facilitate sustained communication and feedback loops between researchers, policymakers, and implementers across sectors, strengthening ownership and accountability for policy uptake. By embedding evidence translation within existing governance and planning frameworks, KTPs enhance national capacity for evidence-informed decision making, improve the timeliness and coherence of AMR policy responses, and support continuity despite political or funding transitions. In the absence of a dedicated KTP, evidence brief development and policy engagement efforts risk remaining fragmented, inconsistent, and difficult to sustain, ultimately limiting their impact on AMR control [9,10,54,56,57,58].

While RADAAR Phase II focused on selected core countries, the methodology and training model developed are adaptable and scalable. The framework may be applied by other low- and middle-income countries in Africa and Asia to strengthen evidence-to-policy translation for AMR and other public health priorities, subject to future funding and country engagement.

## 4. Methods: The RADAAR Approach to Bridging the Evidence-to-Policy Gap

The RADAAR project provided a structured and context sensitive methodology to strengthen evidence-to-policy mechanisms for AMR in LMICs. The approach focused on embedding evidence-informed decision making within existing national AMR policy systems rather than treating evidence use as a standalone or one-off activity. Participating countries received support to identify and prioritize context-specific AMR issues, review and synthesize available local and global evidence, and develop tailored EBPs.

A central component of the strategy was capacity building through integrated training, mentorship, and multisectoral engagement across human, animal, food, and environmental health sectors. Structured policy dialogues were conducted to guide discussion on evidence-based policy options and foster high-level political commitment [59]. The RADAAR approach combined these dialogues with technical training and active stakeholder participation to integrate evidence-informed decision making into routine AMR policy development, moving beyond isolated data collection activities.

### 4.1. Intervention Framework and Implementation Approach

This collaborative initiative involved the RADAAR project, IVI, Seoul, Republic of Korea; the Evidence-Informed Policy Network (EVIPNet), a World Health Organization (WHO) initiative, Geneva, Switzerland; and the Knowledge to Policy (K2P) Center at the American University of Beirut, a WHO Collaborating Centre for Policy and Practice, Beirut, Lebanon. Implementation followed an integrated delivery model, combining hybrid learning formats, tailored technical assistance, and country-led policy dialogues, anchored in a One Health approach to support coordinated multisectoral engagement.

This delivery model was built on lessons from a fully online pilot conducted in 2021 under RADAAR Phase I. While the pilot showed feasibility, it highlighted the need for a more interactive and participatory approach with opportunities for in-person engagement [15]. These lessons directly shaped the design of the current project design, incorporating an in-person intensive workshop followed by online follow-up, and shifting to fully online engagement in contexts where in-person participation was not possible.

### 4.2. Participant Country Selection

Country selection was guided by predefined project criteria, including existing collaborative relationships with IVI to facilitate coordination, effective communication, and balanced regional representation from Asia and Africa, all within Fleming Fund-supported countries [21]. At the country level, selection also aimed to ensure multisectoral participation, with representation from ministries of health, agriculture, environment, and food safety, as well as research institutions, public health laboratories, NGOs, and development partners. Each country established a core working group and a steering committee to provide oversight. This multi-sectoral composition ensured effective engagement of key decision makers across relevant sectors.

Based on the selection criteria, Bhutan, Ghana, Kenya, and Lao PDR were identified as focus countries for RADAAR Phase II. Similar to the focus countries in Phase I, namely Bangladesh, Malawi, Nepal, and Uganda, these Phase II priority countries received more intensive technical assistance reflecting their regional representation and demonstrated engagement in AMR policy processes and readiness to integrate RADAAR support within national strategies.

**Figure 2:**
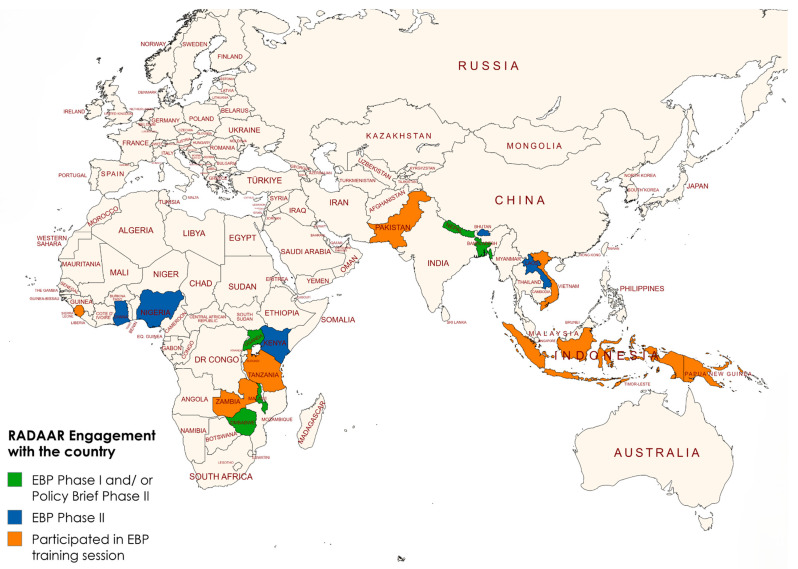
Selected countries in Asia and Africa engaged in the RADAAR initiative and scope of work (This figure was generated using the World Map—Simple template from MapChart Version 7.2.1-2) [60].

In addition to the Phase II focus countries, RADAAR extended support to 14 other Fleming Fund priority countries through a phased engagement model. This included targeted support for EBPs development in Nigeria and policy brief development in Bangladesh, Nepal, Uganda, and Zimbabwe, addressing nationally prioritized AMR issues. The remaining nine countries participated primarily in EBP training sessions. Although Tanzania, Uganda, and Zimbabwe expressed interest in advancing to full EBP development and initiated the process during the training sessions, they were unable to continue beyond the initial stages. Overall, 18 Fleming Fund countries engaged in RADAAR activities, including a lecture series on evidence-based policy and policy brief development, contributing to broader regional capacity strengthening, as illustrated in Figure 2.

### 4.3. The EBP Framework

The methodology followed an adapted EVIPNet knowledge translation framework which included the following steps [61]:

1. Identifying current AMR-related issues, followed by systematic prioritization of a single issue by consensus among multisectoral stakeholders.
2. Providing sufficient evidence to support the issue, inform policy options, and guide implementation considerations.
3. Developing an EBP on the prioritized issue using a structured format developed by WHO-EVIPNet to convert complex AMR data into accessible policy-ready briefs.
4. Involving multisectoral stakeholders throughout the process to validate and refine the EBP, ensuring buy-in for the policy options identified and developed.
5. Facilitating policy dialogues to promote the uptake of evidence-based recommendations.

### 4.4. Training Design and Delivery

**Figure 3:**
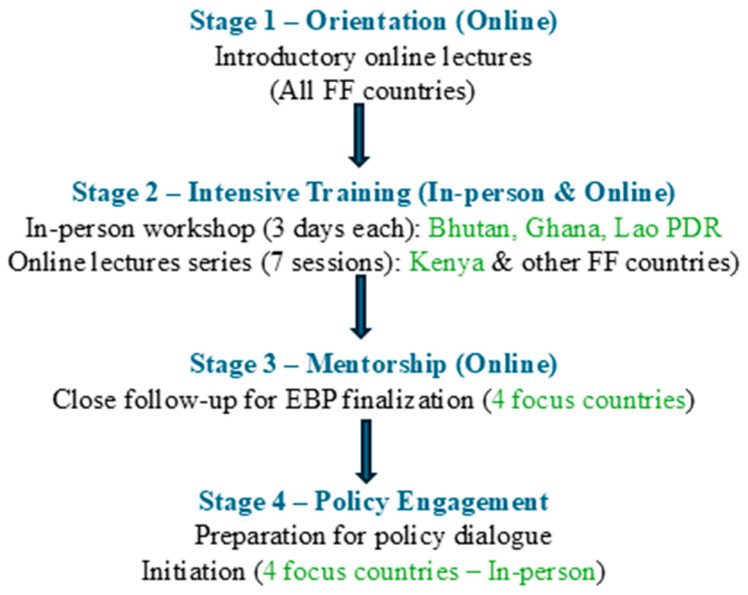
RADAAR project workflow: From policy brief development to policy dialogue.

To operationalize this approach in practice, four stages were designed to guide the development of the EBP across the selected FF countries (Figure 3). The process began in October 2024 with an introductory lecture and concluded in January 2026 with the completion of policy dialogues in the four focus countries.

***Stage 1***—Orientation (Online): In October 2024, an online introductory session was conducted for all FF priority countries to establish a shared foundation in evidence-informed policymaking. Stakeholders from 18 FF priority countries participated in the lectures introducing the EBP approach, its key steps, and expected outcomes.

***Stage 2***—Intensive Training (Hybrid): This was followed by intensive training delivered through two complementary modalities covering the same seven modules (Table 3). First, three day in-person workshops were held in Bhutan in October 2024, Ghana in February 2025, and Lao PDR in December 2024. Although the initial plan for Kenya involved a three-day in-person training, this was cancelled due to logistical and administrative constraints. In addition, several non-focus countries expressed interest in participating in the process. As a result, the training was delivered online to ensure broader accessibility beyond Kenya. Subsequently, Kenya and other FF countries participated in a seven-session online lecture series in June and July 2025, with each session lasting approximately three hours. Together, these approaches ensured consistent delivery of technical content across all participating countries, regardless of format. In addition, video recordings, presentation slides, and all templates and tools were made available to all FF priority countries, including the four focus countries.

***Stage 3***—Mentorship (Online): The four focus countries received structured online mentorship to support EBP development until completion. Additional on-demand support was provided to the other participating countries as needed. Mentorship was provided to Bhutan from October 2024 to August 2025, Ghana from February 2025 to December 2025, Lao PDR from December 2024 to December 2025, and Kenya from July 2025 to January 2026.

***Stage 4***—Policy Engagement: In-person policy dialogue events were conducted in the four focus countries to facilitate engagement with decision makers and support the use of EBPs. These dialogues took place in Bhutan in August 2025, Ghana in December 2025, and Kenya and Lao PDR in January 2026.

This approach was designed to build participants’ capacity to systematically frame AMR problems, identify evidence-supported policy options, consider implementation challenges, and present EBPs in accessible formats, while fostering sustained stakeholder engagement.

### 4.5. Process

**Figure 4:**
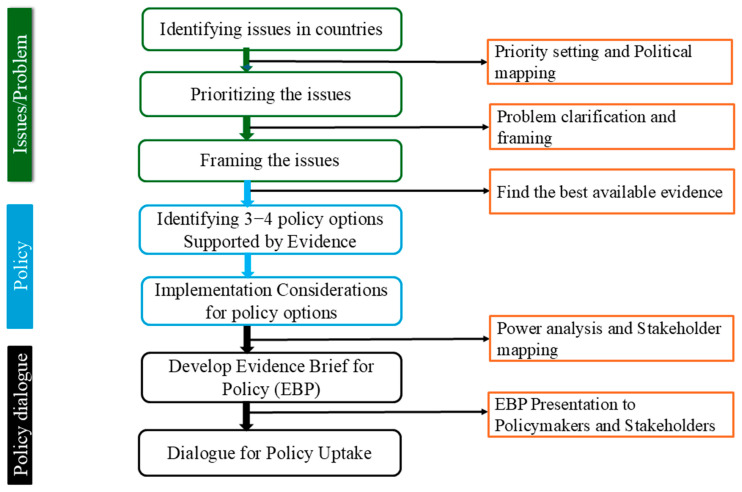
Structured process for evidence-informed policymaking.

Figure 4 outlines the structured WHO-EVIPNet process for evidence-informed policymaking. Following identification and prioritization of AMR issues, each country framed a single prioritized issue and gathered supporting evidence from national data, which was supplemented by regional or global evidence when needed.

Three to four policy options were then developed in every country-specific EBP, each with implementation considerations, anticipated benefits, and potential consequences of inaction. Every stage of the process was evidence based, drawing primarily on the published literature, particularly systematic reviews, reports, and, where relevant, the gray literature from countries.

Each of the four focus countries established a core working group working closely with the RADAAR team through targeted mentorship and technical guidance. This collaborative structure enabled countries to independently develop their EBP tailored to national AMR priorities and contextual challenges (Table 4).

Finally, after finalizing the EBP, the summary of the EBP was presented to high-level stakeholders and decision makers as part of the policy dialogue process.

For the purposes of this project, successful evidence brief for policy was defined as the development and completion of a structured evidence brief that followed all prescribed steps and procedures, aligned with nationally prioritized AMR issues, and culminated in its presentation during the planned policy dialogue phase with relevant multisectoral stakeholders. Within this framework, a proper policy dialogue was defined as a structured exchange between technical experts and decision makers that generated documented feedback, consideration of endorsement, or identification of concrete pathways for integration into policy processes.

## 5. Conclusions

Bridging the gap between AMR data generation and policy uptake remains urgent. While surveillance systems and research initiatives have produced valuable data, much of this evidence remains underutilized, reducing its potential to inform effective and timely health interventions.

The RADAAR initiative demonstrates that strengthening national capacity for evidence-informed decision making requires more than data availability alone. Institutionalized knowledge translation mechanisms, sustained technical mentorship, structured policy dialogues, and meaningful multisectoral engagement are critical to converting evidence into actionable and nationally owned policy responses. By embedding these approaches within existing governance systems and applying a One Health lens, the RADAAR initiative offers a practical and scalable model for strengthening evidence-to-policy pathways.

As AMR and other pressing public health threats continue to evolve, ensuring that high-quality evidence informs decision making is not only a technical necessity but a strategic imperative for sustainable, coordinated, and effective public health action.

## Figures and Tables

**Table 1:** Comparison of survey results of pre- and post-training modules.

Thematic Areas	Pre-Training Survey	Post-Training Survey (Immediate Impact)
**Multisector engagement**	87% (37/43) of countries reported involvement	91.7% of participants strongly agreed on value
**Confidence and skills**	100% (43/43) were familiar with policy briefs, but not specifically with evidence-based policy briefs	94.4% of participants were confident in applying skills
**Policy brief accessibility (experts and non-experts)**	39.5% (17/43) accessible to non-experts	Increased awareness of accessibility gaps and recognition of the need to adapt briefs for non-expert audiences
**Government endorsement of policy brief**	34.9% (15/43) endorsed by government	Increased recognition of the importance of government endorsement and proactive engagement with decision makers
**Dissemination of outcome**	30.2% (13/43) shared beyond internal stakeholders	Greater understanding of the need for broader dissemination, though external sharing remained limited at this stage
**Recommendations used**	51.2% (22/43) used in decision making	63.9% reported opportunities to apply the recommendations in decision making

**Table 2:** Challenges, lessons learned, and recommendations for strengthening AMR evidence-to-policy translation.

Theme	Challenges	Lessons Learned	Recommendations
**Multisectoral coordination**	Sectoral fragmentation, diverse priorities	Inclusive engagement enhances ownership	Co-develop EBPs with multisector input
**Capacity**	Data analysis not linked to policy	Hybrid training effective with mentorship	Targeted capacity building for policymakers
**Sustainability**	Limited funding, competing priorities	Regional/global partnerships amplify efforts	Ensure continuous funding, leverage networks
**Research–policy linkage**	Disconnect between outputs and needs	Structured dialogues boost uptake	Establish knowledge translation platform

**Table 3:** Capacity-building program: Seven-module training on evidence-informed policymaking for AMR.

Module	Topic	Learning Objectives
1	Introduction to Evidence-Informed Policymaking and the Policy Process	Covers EIP foundations, policy models, and core components of an EBP
2	Developing an EBP: Problem Clarification and Framing Part I: Priority setting and identifying issues	Introduces structured tools for selecting and defining national AMR policy priorities
3	Developing EBPs: Problem Clarification and Framing Part II: Framing the issues with evidence	Focuses on problem-framing techniques using behavioral and policy frameworks
4	Finding the Best Available Evidence	Provides practical training on search design, database navigation, and quality appraisal
5	Developing Policy Options	Covers constructing problem statements, identifying policy options, and synthesizing evidence
6	Closing the Loop: From Policy Options to Implementation Considerations	Focus on refining the EBPs, policy options, implementation considerations, and stakeholder mapping
7	Communicating the EBP: Visualization and the Role of Media	Guides teams on evidence use, communication strategies, knowledge uptake, and policy dialogue preparation

**Table 4:** Country and title of corresponding evidence brief for policy.

Country	Topic
**Bhutan**	Combating Antimicrobial Resistance through the Control of Healthcare-Associated Infection in Bhutan [16]
**Ghana**	Strengthening AMR Surveillance Systems using One Health Approach in Ghana [17]
**Kenya**	Strengthening Kenya’s Response to Antimicrobial Resistance through Sustainable Financing [18]
**Lao PDR**	Mitigating AMR-related Mortality and Morbidity in Lao PDR through Enhanced Awareness and Education of the Public and Health Care Providers in Both Human and Animal Health Sectors [19]

## Data Availability

All data underlying the results are available as part of the article, and no additional source data is required.

## Abbreviations

AMR	Antimicrobial resistance
LMICs	Low and middle-income countries
RADAAR	Regional AMR Data Analysis for Advocacy, Response, and Policy
EBP	Evidence brief for policy
E2P	Evidence to policy
K2P	Knowledge to policy
EVIPNet	Evidence-Informed Policy Network
TRACSS	Tracking AMR Country Self-Assessment Survey
KTP	Knowledge translation platform
NGOs	Non-governmental organizations
INGOs	International non-governmental organizations
FF	Fleming Fund
WHO	World Health Organization
IVI	International Vaccine Institute
Lao PDR	Lao People’s Democratic Republic
GLASS	Global AMR and Use Surveillance System
